# Expression of ceramide-metabolising enzymes in subcutaneous and intra-abdominal human adipose tissue

**DOI:** 10.1186/1476-511X-11-115

**Published:** 2012-09-13

**Authors:** Maria Kolak, Joanna Gertow, Jukka Westerbacka, Scott A Summers, Jan Liska, Anders Franco-Cereceda, Matej Orešič, Hannele Yki-Järvinen, Per Eriksson, Rachel M Fisher

**Affiliations:** 1 Atherosclerosis Research Unit, Department of Medicine (Solna), Center for Molecular Medicine, Karolinska Institutet, Stockholm, Sweden; 2 Division of Diabetes, Department of Medicine, University of Helsinki, Helsinki, Finland; 3 Program in Cardiovascular and Metabolic Diseases, Duke-National University of Singapore Graduate Medical School, Singapore; 4 Cardiothoracic Surgery Unit, Department of Molecular Medicine and Surgery, Karolinska Institutet, Stockholm, Sweden; 5 VTT Technical Research Centre of Finland, Espoo, Finland

**Keywords:** Adipose tissue, Ceramide, Human, Inflammation, Sphingomyelinase

## Abstract

**Background:**

Inflammation and increased ceramide concentrations characterise adipose tissue of obese women with high liver fat content compared to equally obese women with normal liver fat content. The present study characterises enzymes involved in ceramide metabolism in subcutaneous and intra-abdominal adipose tissue.

**Methods:**

Pathways leading to increased ceramide concentrations in inflamed versus non-inflamed adipose tissue were investigated by quantifying expression levels of key enzymes involved in ceramide metabolism. Sphingomyelinases (sphingomyelin phosphodiesterases SMPD1-3) were investigated further using immunohistochemistry to establish their location within adipose tissue, and their mRNA expression levels were determined in subcutaneous and intra-abdominal adipose tissue from both non-obese and obese subject.

**Results:**

Gene expression levels of sphingomyelinases, enzymes that hydrolyse sphingomyelin to ceramide, rather than enzymes involved in *de novo* ceramide synthesis, were higher in inflamed compared to non-inflamed adipose tissue of obese women (with high and normal liver fat contents respectively). Sphingomyelinases were localised to both macrophages and adipocytes, but also to blood vessels and to extracellular regions surrounding vessels within adipose tissue. Expression levels of SMPD3 mRNA correlated significantly with concentrations of different ceramides and sphingomyelins. In both non-obese and obese subjects SMPD3 mRNA levels were higher in the more inflamed intra-abdominal compared to the subcutaneous adipose tissue depot.

**Conclusions:**

Generation of ceramides within adipose tissue as a result of sphingomyelinase action may contribute to inflammation in human adipose tissue.

## Introduction

Low grade systemic inflammation and insulin resistance often occur together, and adipose tissue is a site of inflammation in the insulin resistant state. In adipose tissue of obese and insulin resistant subjects macrophage number and inflammatory cytokine production are increased, while adiponectin production is decreased [1]. The mechanisms causing macrophage recruitment into adipose tissue are unclear, although adipocyte size has been implicated [1]. Furthermore, macrophages are often found clustered in “crown-like structures” surrounding individual adipocytes [2,3], which appear to be dead [2], suggesting that dead/dying adipocytes may trigger macrophage influx, but the reason for adipocyte death is unknown. Another theory is that hypoxia may develop in regions of obese adipose tissue distant from the vasculature, causing hypoxic adipocytes to produce inflammatory cytokines and/or to die thus trig-gering macrophage infiltration [4]. An interesting possibility is that there are similarities between inflammatory pathways in obese/insulin resistant adipose tissue and the atherosclerotic artery wall. For example, oxidized lipid epitopes within adipose tissue might attract macrophages, or particular lipids (or their metabolites) might interfere with signalling, but this remains to be established.

The degree of insulin resistance, rather than the degree of obesity, is of critical importance for the inflammatory status of adipose tissue [5]. Since fat accumulation in the liver predicts insulin resistance better than obesity [6], we postulated previously that hepatic fat content might distinguish between obese subjects who develop insulin resistance and adipose tissue inflammation and those who do not. This hypothesis was supported by investigation of adipose tissue from obese women with differing liver fat content [3]. We found greater expression of macrophage-related genes and lower expression of adiponectin in subcutaneous adipose tissue from obese women with a high liver fat content compared to obese women with a normal liver fat content, but with comparable degrees of obesity. Furthermore, concentrations of certain ceramides and sphingomyelins were higher in the inflamed adipose tissue of women with greater hepatic fat content [3].

Ceramides act as second messengers in numerous signalling pathways involved in insulin action, inflammation, angiogenesis and cell death [7,8]. Ceramides are increased in serum, skeletal muscle and liver of obese rodents and humans (reviewed in [9]), correlate negatively with insulin sensitivity [10-12] and positively with circulating IL6 [13] thereby implicating ceramides in the development of insulin resistance and inflammation. Indeed, it was recently shown that a lowering of cellular ceramide mediates adiponectin’s beneficial metabolic effects [12] and that ceramide links lipid-induced inflammatory pathways to the development of insulin resistance [14]. Data on adipose tissue ceramides, which are more limited and almost entirely restricted to rodent models, link increased ceramides or ceramide derivatives in this tissue to obesity, insulin resistance and local inflammation [15-20]. Therefore in the current study we investigate expression and localisation in human adipose tissue of enzymes involved in ceramide metabolism, with a particular focus on sphingomyelinases, to understand better the mechanisms underlying ceramide production in human adipose tissue since this is a largely unexplored area.

## Methods

### Study populations

Subcutaneous adipose tissue biopsies were collected from 20 obese, but otherwise healthy women (age 30–59 years, BMI 30–42 kg/m^2^), recruited at Helsinki University Central Hospital, Finland [3]. The women were divided into two groups according to their liver fat content as measured by magnetic resonance proton spectroscopy: normal liver fat (n = 10, liver fat range 1–3.5%) and high liver fat (n = 10, range 6-35%). These groups were matched for age, BMI and subcutaneous and intra-abdominal fat masses as determined by MRI. However, compared to the normal liver fat group, the high liver fat group had higher concentrations of ceramide and sphingomyelin in subcutaneous adipose tissue and this tissue was more inflamed, as assessed by significantly higher expression levels of CD68, CCL2, CCL3 and PAI-1, and significantly lower expression levels of adiponectin and PPARG [3].

A second group consisted of 23 non-obese patients undergoing heart-valve surgery at Karolinska University Hospital, Stockholm, Sweden, without documented coronary artery disease. Biopsies were obtained at surgery from subcutaneous and intra-abdominal adipose tissue, and from the liver. Blood samples were taken after overnight fast. Clinical characteristics are summarized in Table 1. In addition, subcutaneous and intra-abdominal biopsies were taken at surgery from 8 morbidly obese patients (2 women, 6 men, age 45 ± 2 years, BMI 52.6 ± 2.0 kg/m^2^) undergoing laparoscopic gastric bypass operation at Helsinki University Central Hospital.

**Table 1:** Clinical characteristics of patients undergoing heart-valve surgery

	**Males**	**Females**
	**n = 14**	**n = 9**
Age (years)	64.8 ± 3.9	68.8 ± 3.3
BMI (kg/m^2^)	27.2 ± 1.0	26.5 ± 1.3
Glucose (mmol/l)	5.0 ± 0.1	5.2 ± 0.2
HbA1c (%)	4.4 ± 0.1	4.7 ± 0.1*
Insulin (pmol/l)	56.2 ± 10.5	49.6 ± 8.6
Triacylglycerol (mmol/l)	1.2 ± 0.2	0.9 ± 0.1
Total-cholesterol (mmol/l)	5.1 ± 0.2	4.7 ± 0.4
LDL-cholesterol (mmol/l)	3.4 ± 0.2	2.7 ± 0.3
HDL-cholesterol (mmol/l)	1.2 ± 0.1	1.6 ± 0.2*
ALAT (μkat/l)	0.50 ± 0.04	0.41 ± 0.04
γGT (μkat/l)	0.45 ± 0.11	0.48 ± 0.20
CRP (mg/l)	2.9 ± 0.6	2.6 ± 1.0

* P < 0.05 for differences between the groups. Values are expressed as mean ± SEM. *ALAT*: alanine aminotransferase; γ*GT*: gamma-glutamyl transferase; *CRP*: C-reactive protein.

The nature and potential risks of the study were explained to all subjects before obtaining written informed consent. Study protocols were approved by the ethics committees of Karolinska Institutet and Helsinki University Central Hospital.

### Adipose tissue biopsies

Tissue for gene expression analysis (approximately 150 mg) was either frozen in liquid nitrogen and kept at −80°C prior to RNA extraction, for biopsies taken from obese subjects in Helsinki, or fixed in RNA-later (Ambion), for biopsies taken from patients undergoing heart-valve surgery in Stockholm. A part of each biopsy (from the 20 obese women and the 23 patients under-going heart-valve surgery) was fixed in 4% zinc formaldehyde for subsequent paraffin embedding and immunohistochemistry. Tissue from the 20 obese women that had been frozen in liquid nitrogen and preserved at −80°C was used for lipidomic analysis, as described pre-viously [3]. In brief, adipose tissue lipids were extracted using chloroform:methanol (2:1) solvent and analysed using Ultra Performance Liquid Chromatography coupled to time-of-flight mass spectrometry (UPLC-QTOFMS). A total of 154 molecular lipids were measured including the following sphingolipids: ceramides Cer(d18:0/22:0), Cer(d18:1/16:0) and Cer(d18:1/24:1); and sphingomyelins SM(d18:1/16:0), SM(d18:1/18:0), SM(d18:1/20:0), SM(d18:1/22:0), SM(d18:1/22:1), SM(d18:1/24:1) and SM(d18:1/24:2). All the identified lipids were quantified by calibrating with corresponding class-specific internal standards. Sphingomyelins were calibrated with phosphatidylcholine PC(17:0/17:0).

### RNA isolation and cDNA synthesis

For biopsies collected in Sweden, approximately 150 mg of adipose tissue was homogenized in Trizol (Invitrogen) using the Fastprep Homogenizer (Qbiogene). RNA purification, including DNase treatment, was performed using RNeasy mini kits (Qiagen) according to the manufacturer’s protocol. RNA concentrations were measured using a NanoDrop spectrophotometer (Thermo). RNA quality was analyzed by an Agilent Bioanalyzer 2100 (Agilent Technologies). Isolated RNA was stored at −80°C until cDNA synthesis. A total of 1 μg RNA was transcribed into cDNA using Superscript III reverse transcriptase (Invitrogen) and oligo (dT)_12–18_ primer. RNA isolation and cDNA synthesis from biopsies taken in Finland was described previously [3].

### Quantification of gene expression

mRNA expression of specific genes was quantified by real time PCR using the ABI 7000 Sequence Detection System instrument and software (Applied Biosystems). cDNA synthesized from 15 ng of total RNA was mixed with TaqMan Universal PCR Master Mix (Applied Biosystems) and a gene-specific primer and probe mixture (pre-developed TaqMan Gene Expression Assays, Applied Biosystems) in a final volume of 25 μl. The assays used were: Hs00154355_m1 for CD68, Hs00234140_m1 for CCL2, Hs00234142_m1 for CCL3, Hs00605917_m1 for adiponectin, Hs00174131_m1 for IL6, Hs00174128_m1 for TNFα, Hs99999910_m1 for TBP, Hs99999902_m1 for RPLP0 and assays shown in Table 2. All samples were run in duplicate. Relative expression levels were determined using a 5-point serially diluted standard curve, generated from cDNA from human adipose tissue. Gene expression was expressed in arbitrary units and normalized relative to the housekeeping genes RPLP0 and TBP to compensate for differences in cDNA loading. The average of these two values was used for normalization.

**Table 2:** Expression in subcutaneous adipose tissue of ceramide-metabolising enzymes in relation to adipose tissue inflammation

**Gene**	**ABI assay ID**	**Ct value**	**Less inflamed adipose tissue, normal liver fat**	**More inflamed adipose tissue, high liver fat**	***P***
			**n = 10**	**n = 10**	
SPTLC1	Hs00272311_m1	27.2	0.99 ± 0.04	1.05 ± 0.06	0.20
SPTLC2	Hs00191585_m1	26.8	0.97 ± 0.07	1.00 ± 0.05	0.39
DEGS1	Hs00186447_m1	25.9	0.99 ± 0.05	1.07 ± 0.05	0.11
LASS1	Hs00242151_m1	33.2	9.07 ± 3.12	9.22 ± 1.63	0.48
LASS4	Hs00226114_m1	27.1	1.08 ± 0.10	1.07 ± 0.16	0.49
LASS6	Hs00826756_m1	28.8	0.87 ± 0.07	0.89 ± 0.07	0.42
ASAH1	Hs00602774_m1	24.3	1.16 ± 0.10	1.47 ± 0.13	**0.03**
UGCG	Hs00234293_m1	28.2	0.91 ± 0.08	0.96 ± 0.06	0.34
SGMS1	Hs00380453_m1	28.0	1.01 ± 0.05	1.10 ± 0.06	0.12
SGMS2	Hs00398067_m1	30.6	2.09 ± 0.27	2.57 ± 0.32	0.13
SMPD1	Hs00609415_m1	26.6	1.51 ± 0.10	1.85 ± 0.10	**0.01**^a^
SMPD2	Hs00162006_m1	29.8	1.13 ± 0.06	1.26 ± 0.06	0.08 ^a^
SMPD3	Hs00218713_m1	33.8	1.33 ± 0.13	1.76 ± 0.18	**0.05**^a^
SMPD4	Hs00215775_m1	29.1	1.57 ± 0.09	1.52 ± 0.09	0.35
CERK	Hs00368483_m1	29.3	1.57 ± 0.15	1.87 ± 0.10	0.06
SPHK1	Hs00184211_m1	28.9	12.18 ± 1.49	17.64 ± 1.65	**0.01**
CGT	Hs00409961_m1	35.5	4.45 ± 1.29	3.45 ± 0.52	0.24
CDH5	Hs00174344_m1	28.3	1.16 ± 0.14	1.43 ± 0.13	0.09
SELE	Hs00950401_m1	33.2	0.72 ± 0.15	2.45 ± 0.94	**0.04**
VEGF	Hs0090054_m1	26.4	4.13 ± 0.37	3.71 ± 0.52	0.25
HIF-1α	Hs00153153_m1	27.6	1.39 ± 0.12	1.65 ± 0.08	**0.02**

Relative gene expression levels of genes involved in ceramide and sphingomyelin metabolism in subcutaneous adipose tissue of obese women with different degrees of adipose tissue inflammation are given. *SPTLC*: serine palmitoyl transferase long-chain; *DEGS*: dihydroceramide desaturase; *LASS*: LAG1 homolog (ceramide synthase); *ASAH*: N-acylsphingosine amidohydrolase (ceramidase); *UGCG*: UDP-glucose ceramide glucosyltransferase; *SGMS*: sphingomyelin synthase; *SMPD*: sphingomyelin phosphodiesterase (sphingomyelinase); *CERK*: ceramide kinase; *SPHK*: sphingosine kinase; *CGT*: ceramide glycosyl transferase; *CDH5*: cadherin 5 (vascular endothelium); *SELE*: selectin E; *VEGF*: vascular endothelial growth factor; *HIF-1α*: hypoxia-inducible factor 1α. Gene expression was normalized to housekeeping genes RPLP0 and TBP, which did not differ between the groups (for *RPLP0*: 1.94 ± 0.26 vs 2.11 ± 0.17, NS; for *TBP*: 3.96 ± 0.48 vs 3.97 ± 0.44, NS). Values are expressed as mean ± SEM. ^a^ Reported previously [3].

### Liver biopsies

RNA extraction, gene expression analysis with Affymetrix GeneChip Human Exon 1.0 ST arrays and normalisation were performed as described [21]. Of the 23 patients undergoing heart-valve surgery included in the present study, liver data were available from 21. Hepatic expression levels of ACSL4, DGAT2, PNPLA3 and PPARG were selected for subsequent analysis.

### Immunohistochemistry

Five μm thick serial sections were deparaffinized and boiled in TE-buffer (10 mM Tris-Cl, 1 mM EDTA, pH 7.5) at 98°C for 30 min to demask epitopes. After blocking with normal serum (goat or horse, Vector Laboratories), sections were incubated with primary antibodies at 4°C overnight. Primary antibodies were: anti-SMPD1 sc-9815, 1:50, anti-SMPD2 sc-26212, 1:50, anti-SMPD3 sc-67692, 1:50, anti-PECAM-1 sc-1506, 1:100 (all goat polyclonal IgG, Santa Cruz Biotechnology) and mouse monoclonal anti-CD68, 1:200 (Novocastra Laboratories), anti-ASAH1, 1:50 (Abcam) and anti-apolipoprotein B, 1:50 (H61428M Biosite). After washing in PBS buffer, sections were incubated with secondary biotinylated goat anti mouse, 1:2000 (Dako) or biotinylated horse anti goat, 1:2000 (Vector Laboratories) antibodies. Staining was visualized using avidin-biotin peroxidase complex (ABC, Vector Laboratories) followed by 3.3´-diaminobenzidine tetrachloride (DAB, Vector Laboratories). All sections were counterstained with Harris haematoxylin (Histolab). Collagen was stained with Sirius red (Bie & Berntsen) and visualized by polar light microscopy.

### Statistical analysis

The Statview software (SAS Institute Inc, Cary, NC, USA) was used. Physical and biochemical characteristics of the study subjects were analyzed using non-parametric methods. Groups were compared using the Mann–Whitney test. Different adipose tissue depots were compared using the Wilcoxon signed rank test. All correlations were performed using Spearman’s rank correlation. Statistical significance was assigned to a value of *P* < 0.05. Data are presented as mean ± SEM. Hepatic expression levels of selected genes were used to generate a mean standard deviation score for hepatic triacylglycerol accumulation. For each subject, each value was expressed as standard deviations of difference from the population mean. The mean hepatic triacylglycerol accumulation score was calculated as the mean of these standard deviation scores: (ACSL4 + DGAT2 + PNPLA3 + PPARG)/4.

## Results

**Figure 1:**
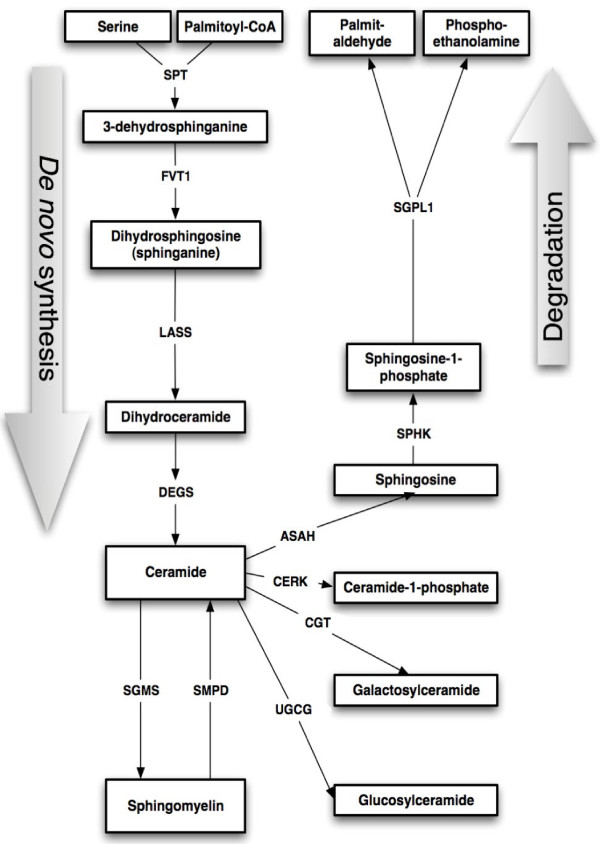
Schematic diagram depicting enzymatic reactions involved in the synthesis and degradation of ceramide and sphingomyelin.

To investigate the basis for elevated ceramide/sphingomyelin concentrations in inflamed adipose tissue, expression levels of genes involved in ceramide synthesis and metabolism (summarised in Figure 1) were quantified in subcutaneous adipose tissue from obese women. These women were divided into two groups based on their liver fat content: normal (2.3 ± 0.3%, n = 10) and high (14.4 ± 2.9%, n = 10). The groups were similar with respect to age, BMI and subcutaneous and intra-abdominal adipose tissue masses, but the high liver fat group had a more unfavourable metabolic profile and more inflammation and higher ceramide and sphingomyelin concentrations in their subcutaneous adipose tissue [3]. Genes involved in *de novo* ceramide synthesis (SPTLC1, SPTLC2, DEGS1, LASS1, LASS4 and LASS6) were not differentially expressed between the groups (Table 2). Ceramide-metabolising enzymes CGT, CERK, SGMS1, SGMS2 and UGCG were also similarly expressed. However, ASAH1 and SPHK1 were expressed at significantly higher levels in the women with more inflamed adipose tissue (*P* = 0.03 and *P* = 0.01, respectively). Expression levels of three sphingomyelinases were greater (*P* = 0.01 for SMPD1, *P* = 0.05 for SMPD3), or tended to be greater (*P* = 0.08 for SMPD2) in the women with high liver fat and more inflammation and higher ceramide/sphingomyelin content in their adipose tissue [3], but expression of a fourth sphingomyelinase, SMPD4, did not differ.

To establish whether ceramide and sphingomyelin metabolising enzymes that displayed differential mRNA expression between the two groups, namely SMPD1, SMPD2, SMPD3, ASAH1 and SPHK1, might be determinants of ceramide and/or sphingomyelin concentrations in adipose tissue, their mRNA levels were correlated with these lipid species (Table 3). Expression levels of SMPD3 in adipose tissue were statistically significantly correlated with 2 out of the 3 ceramides and 6 out of the 7 sphingomyelins that were quantified with the lipidomic analysis. SPHK1 expression was significantly correlated with one ceramide and two sphingomyelin species.

**Table 3:** Correlations between sphingomyelinase, ceramidase and sphingosine kinase gene expression, and ceramide and sphingomyelin concentrations in subcutaneous adipose tissue

	**SMPD1**		**SMPD2**		**SMPD3**		**ASAH1**		**SPHK1**	
	**r**	***P***	**r**	***P***	**r**	***P***	**r**	***P***	**r**	***P***
Cer(d18:0/22:0)	0.380	0.097	−0.170	0.459	0.244	0.288	0.005	0.984	0.259	0.260
Cer(d18:1/16:0)	0.065	0.778	0.089	0.699	**0.498**	**0.030**	0.047	0.839	0.361	0.116
Cer(d18:1/24:1)	0.408	0.076	0.283	0.218	**0.552**	**0.016**	0.344	0.133	**0.526**	**0.022**
SM(d18:1/16:0)	0.135	0.555	0.020	0.932	0.211	0.359	−0.310	0.177	−0.203	0.376
SM(d18:1/18:0)	0.281	0.220	0.411	0.074	**0.496**	**0.031**	−0.164	0.475	0.215	0.349
SM(d18:1/20:0)	0.398	0.082	0.376	0.101	**0.555**	**0.016**	−0.338	0.140	**0.492**	**0.032**
SM(d18:1/22:0)	0.331	0.149	0.356	0.120	**0.595**	**0.009**	0.075	0.743	**0.474**	**0.039**
SM(d18:1/22:1)	0.415	0.070	0.290	0.206	**0.582**	**0.011**	−0.081	0.723	0.445	0.052
SM(d18:1/24:1)	0.253	0.271	0.423	0.066	**0.567**	**0.014**	0.167	0.467	0.289	0.208
SM(d18:1/24:2)	0.329	0.151	0.319	0.165	**0.513**	**0.025**	−0.033	0.885	0.405	0.078

Correlations between gene expression levels of SMPD1-3 (sphingomyelinases), ASAH1 (ceramidase) and SPHK1 (sphingosine kinase) and concentrations of various ceramides (cer) and sphingomyelins (SM) in subcutaneous adipose tissue of obese women (n = 20). Spearman rank correlation coefficients and significance levels are shown. Comparison of sphingolipid concentrations (mean ± SEM, nmol/g adipose tissue) in subcutaneous adipose tissue from women with less inflamed adipose tissue/normal liver fat (n = 10) versus more inflamed adipose tissue/high liver fat content (n = 10), as reported previously [3]: Cer(d18:0/22:0): 3.80 ± 0.58 vs 6.21 ± 1.11, *P* = 0.076; Cer(d18:1/16:0): 2.66 ± 0.28 vs 3.12 ± 0.23, *P* = 0.219; Cer(d18:1/24:1): 5.36 ± 0.47 vs 7.97 ± 0.70, *P* = 0.006; SM(d18:1/16:0): 36.86 ± 2.64 vs 35.10 ± 2.64, *P* = 0.643; SM(d18:1/18:0): 4.88 ± 0.40 vs 6.02 ± 0.46, *P* = 0.077; SM(d18:1/20:0): 5.84 ± 0.50 vs 7.30 ± 0.52, *P* = 0.055; SM(d18:1/22:0): 11.38 ± 0.96 vs 15.38 ± 1.15, *P* = 0.015; SM(d18:1/22:1): 7.13 ± 0.54 vs 8.54 ± 0.67, *P* = 0.121; SM(d18:1/24:1): 17.00 ± 1.33 vs 22.96 ± 1.70, *P* = 0.013; SM(d18:1/24:2): 7.42 ± 0.56 vs 9.69 ± 0.76, *P* = 0.028. Lipidomic analysis was described previously [3].

**Figure 2:**
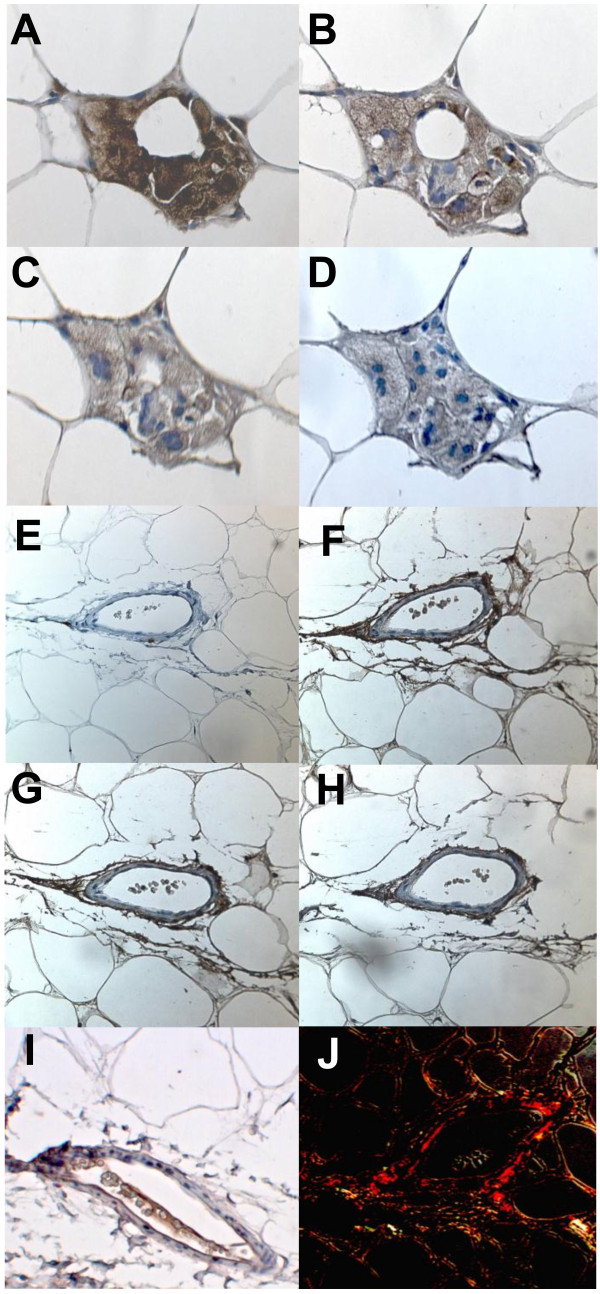
**Localisation of sphingomyelinases in subcutaneous adipose tissue.** Representative pictures of immunohistochemical staining of macrophages and multinuclear giant cells (aggregated macrophages) (**A**-**D**) and a small blood vessel (**E**-**J**) within subcutaneous adipose tissue from an obese woman. Brown coloration indicates macrophage-specific CD68 (**A** and **E**), SMPD1 (**B** and **F**), SMPD2 (**C** and **G**), SMPD3 (**D** and **H**) and PECAM-1 (**I**). All sections were counterstained with haematoxylin (coloured blue). Collagen staining (**J**) was visualized with polarized light to reveal mature, extracellular collagen (coloured red).

Collectively these data suggested a possible role for sphingomyelinase activity in adipose tissue in determining adipose ceramide and/or sphingomyelin concentrations, which may be related to inflammation and macrophage accumulation. To establish the location of these enzymes in adipose tissue, immunohistochemical analysis of subcutaneous adipose tissue from the obese women was performed. SMPD1-3 proteins were expressed in both macrophages and adipocytes within adipose tissue (Figure 2). All three sphingomyelinases were also found in and around blood vessels (Figure 2F-H).

**Figure 3:**
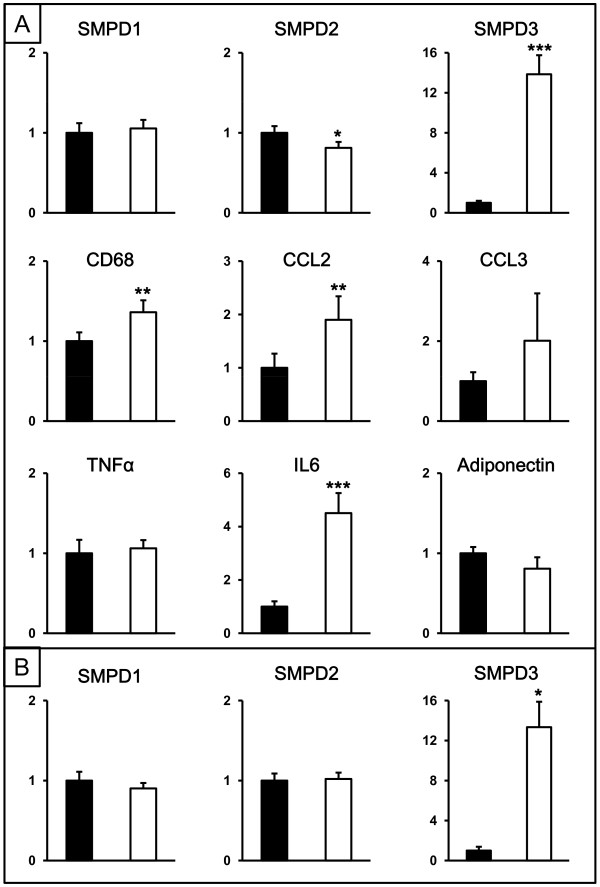
**Gene expression in subcutaneous and intra-abdominal adipose tissue from non-obese and obese individuals.** (**A**) Relative gene expression levels of SMPD1-3, CD68, CCL2, CCL3, TNFα, IL6 and adiponectin in subcutaneous (black bars) and intra-abdominal (white bars) adipose tissue from 23 non-obese individuals. (**B**) Relative gene expression levels of SMPD1-3 in subcutaneous (black bars) and intra-abdominal (white bars) adipose tissue from 8 obese patients. Expression is in arbitrary units normalized to housekeeping genes RPLP0 and TBP, and set to 1 for the subcutaneous depot. * *P* < 0.05, ** *P* <0.01, *** *P* < 0.001 compared to subcutaneous adipose tissue.

**Figure 4:**
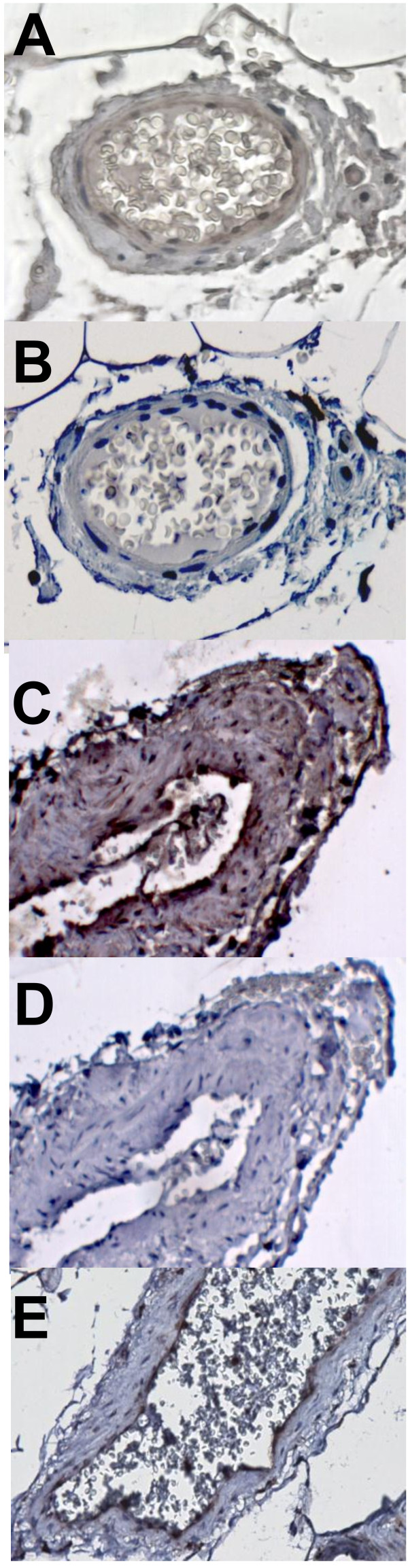
**Localisation of sphingomyelinase SMPD3 and ceramidase ASAH1 in subcutaneous and intra-abdominal adipose tissue.** Representative pictures of immunohistochemical staining of a vessel within subcutaneous (**A**, **B** and **E**) and intra-abdominal (**C** and **D**) adipose tissue from a non-obese individual. Positive staining for SMPD3 (**A** and **C**) and ASAH1 (**E**) is shown as brown coloration. Negative staining is shown in panels **B** and **D**. All sections were counterstained with haematoxylin (coloured blue).

To investigate sphingomyelinases further in human adipose tissue, the mRNA expression and protein distribution of SMDP1-3 was compared in subcutaneous and intra-abdominal adipose tissue from non-obese individuals (clinical characteristics in Table 1). Gene expression levels of the inflammatory markers CD68, CCL2 and IL6 were significantly higher in intra-abdominal compared to subcutaneous fat (Figure 3A). SMPD3 mRNA was higher, while expression of SMPD2 was lower in intra-abdominal compared to subcutaneous fat. Expression of SMPD1 was similar in both depots. Immunohistochemical analysis of subcutaneous and intra-abdominal adipose tissue located SMPD1-3 proteins mainly to blood vessels (Figure 4A and C for SMPD3). Furthermore, an enzyme that metabolises ceramides, ASAH1, was also localized to the vasculature (Figure 4E). In light of our previous data from obese women identifying a relationship between inflammation and ceramide metabolism in adipose tissue and fat accumulation in the liver, we investigated associations between gene expression in adipose tissue and liver in these non-obese individuals. Since direct measurements of liver fat were not available, hepatic mRNA levels of selected genes were used as surrogate markers of liver fat accumulation. Due to the small sample size, only a very limited number of genes were selected: ACSL4, DGAT2, PNPLA3 and PPARG. We have previously shown hepatic expression of ACSL4 and PPARG to be related to liver fat content [22]. DGAT catalyses the committed step in triacylglycerol synthesis with DGAT2 being the dominant DGAT enzyme controlling triacylglycerol homeostasis *in vivo*[23], and PNPLA3 has been implicated in the development of hepatic steatosis [24]. A mean standard deviation score was calculated from the hepatic mRNA expression data for these 4 genes (see methods) to generate a summary score for liver triacylglycerol accumulation. This liver summary score was significantly correlated to expression levels of CD68 (chosen as a marker of macrophage accumulation) in both subcutaneous and intra-abdominal adipose tissue (r = 0.56, *P* = 0.01 and r = 0.49 *P* = 0.03 respectively), but not to adipose tissue sphingomyelinase expression in either depot (SMPD1: r = 0.10, *P* = 0.65 and r = 0.24, *P* = 0.29; SMPD2: r = 0.16, *P* = 0.48 and r = 0.33, *P* = 0.14; and SMPD3: r = 0.10, *P* = 0.65 and r = 0.21, *P* = 0.36 for subcutaneous and intra-abdominal adipose tissue respectively).

Expression patterns of SMPD1-3 mRNA in subcu-taneous and intra-abdominal adipose tissue were also determined in an independent group of 8 morbidly obese subjects (Figure 3B). Levels of SMPD3 mRNA were significantly greater in intra-abdominal than in subcutaneous adipose tissue, but no significant differences were observed for SMPD1 or SMPD2. Differences in gene expression between adipose tissue depots could not be explained by depot-specific differences in house-keeping gene expression and essentially identical results were obtained when non-normalised gene expression data (raw Ct values) were used (data not shown).

**Figure 5:**
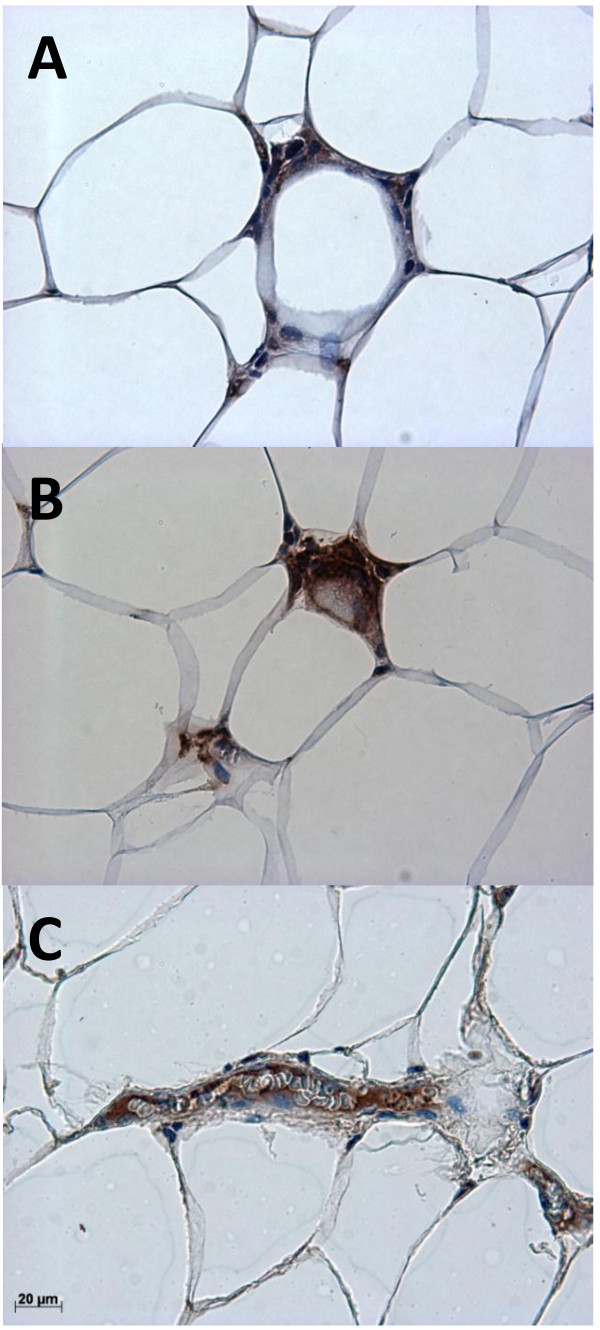
**Localisation of apo B in subcutaneous and intra-abdominal adipose tissue.** Representative pictures of immunohistochemical staining of apo B (**A**, **C**) and CD68 (**B**) in human adipose tissue. (**A**) and (**B**): Serial sections of subcutaneous adipose tissue from an obese woman stained for apo B and CD68 respectively. (**C**): Intra abdominal adipose tissue from a non-obese individual stained for apo B. Positive staining for apo B (**A** and **C**) and CD68 (**B**) is shown as brown coloration. All sections were counterstained with haematoxylin (coloured blue).

Since staining for sphingomyelinases in adipose tissue was strongest in the vicinity of blood vessels, we investigated whether there were differences in adipose tissue vascularity between women with more or less inflammation in their adipose tissue. However, expression levels of a marker of vascular endothelium (CDH5) did not differ between groups (Table 2). Expression levels of markers of hypoxia (HIF-1α) and endothelial activation (E-selectin) were significantly higher in the adipose tissue of women with an increased liver fat content and more inflammation and higher ceramide concentrations in their adipose tissue (Table 2), but these increases in hypoxia and endothelial activation did not appear to be associated with an increase in angiogenesis, since there was no corresponding increase in expression of the marker for endothelial growth, VEGF. In adipose tissue from both obese and non-obese subjects, staining for the integral protein of hepatic lipoproteins, apolipoprotein B (apo B), was found in blood vessels, but also in regions that stained positive for CD68 (Figure 5), suggesting that lipoproteins entering adipose tissue via the vasculature might provide a source of sphingomyelins for hydrolysis by local sphingomyelinases to produce ceramide.

## Discussion

We investigated sphingolipid metabolism in human adipose tissue to identify pathways underlying increased ceramide concentrations in inflamed adipose tissue [3]. Our data suggest that hydrolysis of sphingomyelin to ceramide by sphingomyelinases could explain, at least partly, this increase. Gene expression levels of SMPD3 correlate significantly with concentrations of various ceramides and sphingomyelins in subcutaneous adipose tissue, and are higher in the relatively more inflamed intra-abdominal compared to the subcutaneous depot in both obese and non-obese subjects. Sphingomyelinases are expressed by both adipocytes and macrophages in adipose tissue, but their expression is strongest in and around blood vessels. Our findings implicate a role for sphingomyelinase-mediated generation of ceramide in adipose tissue inflammation.

When comparing inflamed ceramide-rich and relatively less inflamed ceramide-poor subcutaneous adipose tissue of obese women, we report here for the first time that there were no differences in mRNA levels of genes involved in *de novo* ceramide synthesis. However, expression of sphingomyelinases SMPD1 and SMPD3 was significantly higher, while that of SMPD2 tended to be higher in the inflamed adipose tissue group (as reported [3]). Since sphingomyelinases catalyse the conversion of sphingomyelins to ceramide, this pathway rather than *de novo* ceramide synthesis, may underlie the increased ceramide content of the inflamed adipose tissue of these women. Sphingomyelinase activity is increased by oxidative stress both *in vitro *[25] and *in vivo *[26] and sphingomyelinase expression in adipose tissue increases in response to a high fat diet in mouse models [15,27]. Since ceramides stimulate synthesis of pro-inflammatory cytokines by both adipocytes and macrophages [27,28], increased sphingomyelinase activity in adipose tissue could exacerbate the inflammatory milieu and enhance recruitment of macrophages. Therefore investigation of sphingomyelinases in human adipose tissue in relation to inflammation and macrophage accumulation is motivated. To date the only report of sphingomyelinases in human adipose tissue found reduced acid sphingomyelinase, but unchanged neutral sphingomyelinase activity in obese compared to lean patients [29].

Although we observed increased sphingomyelinase mRNA expression in inflamed adipose tissue, sphingomyelinases did not localise only to inflammatory cells (determined by immunohistochemistry). Staining for SMPD1, -2 and −3 was seen in macrophages and also in adipocytes, but the strongest staining was seen in and around blood vessels, the latter being reminiscent of the secretory form of SMPD1 localising to the subendothelial matrix of atherosclerotic lesions [30]. Additionally, the ceramide-metabolising enzyme ASAH1 was found in the vasculature, indicating that blood vessels are important sites for ceramide metabolism within adipose tissue. Indeed, immunohistochemical analysis revealed staining for apo B in areas containing inflammatory cells (positive for CD68) and within blood vessels, indicating access of adipose tissue sphingomyelinases to sphingomyelins within lipoproteins. Previously we found that not only ceramides, but also sphingomyelins were increased in adipose tissue of obese women with more inflamed adipose tissue. The increase in sphingomyelins did not appear to be accounted for by increased local synthesis (as discussed above), but might be explained by an increased delivery of sphingomyelin-rich lipoproteins produced by fatty liver [31], since these women also had increased hepatic fat content, but quantification of lipoprotein delivery to or retention within adipose tissue was not possible in this study. To pursue this idea we investigated the relationship between hepatic triacylglycerol accumulation and adipose tissue ceramide metabolism and inflammation in non-obese individuals. Inflammation in both subcutaneous and intra-abdominal adipose tissue (as assessed by RNA levels of the macrophage marker CD68) was positively related to the expression of genes in the liver reflecting triacylglycerol accumulation, but no such relationships were found for sphingomyelinase expression (SMPD1, -2 or −3) in either adipose tissue depot. This suggests that even in non-obese individuals, the number of macrophages within adipose tissue is linked to hepatic triacylglycerol metabolism. However, sphingomyelinases do not appear to be involved in this relationship in these subjects. Unfortunately no measurements of either adipose tissue ceramide concentrations or liver fat content were available, so no conclusions can be drawn as to the relationship between adipose tissue macrophage accumulation, ceramide concentration and hepatic triacylglycerol content, but our data suggest that sphingomyelinase-mediated generation of ceramide in adipose tissue does not play a major role in this context in non-obese subjects who are unlikely to have fatty livers.

Our data highlighted the potential importance of SMPD3 within adipose tissue in relation to ceramide generation and inflammation for two reasons. Firstly, mRNA levels of SMPD3 correlated significantly with ceramide and sphingomyelin concentrations within adipose tissue of obese women. Secondly, a relatively more inflamed adipose tissue depot, namely intra-abdominal fat, expressed SMPD3 mRNA at significantly greater levels than relatively less inflamed subcutaneous adipose tissue in both non-obese and obese subjects. It is possible that increased SMPD3 activity contributes to the greater ceramide concentrations in intra-abdominal compared to subcutaneous adipose tissue [32]. Adipose tissue hypoxia is proposed to be a major underlying cause for insulin resistance and other disorders associated with obesity, promoting macrophage infiltration and angiogenesis [33]. The location of sphingomyelinases to blood vessels within adipose tissue might indicate their involvement in angiogenesis. The increased expression of markers of hypoxia and endothelial activation, but no differences in markers of either angiogenesis or endothelial cell number in adipose tissue of women with inflamed compared to less inflamed adipose tissue, indicates the existence of hypoxia and an activated endothelium without an apparent decrease in vessel density or onset of angiogenesis. It is possible that the increased degree of inflammation is related to an inability of hypoxia and increased ceramide concentrations to induce angiogenesis within adipose tissue. Since particular importance was assigned to SMPD3 in hypoxic vasoconstriction in the lung [34], SMPD3 may play a role linking hypoxia, ceramide generation and inflammation within adipose tissue, however, this remains to be shown.

The observation that concentrations of some ceramide and sphingomyelin species correlated positively with gene expression levels of SPHK1 (sphingosine kinase) in adipose tissue in the cohort of obese women is also of interest. The product of SPHK1 action is sphingosine-1-phosphate (concentrations of which were not quantified in the present study), a sphingolipid that appears to have the opposite actions to that of ceramide, namely promoting cell survival and proliferation [35]. Indeed, the balance between concentrations of ceramide and sphingosine-1-phosphate is proposed to be an important mechanism controlling cell fate [35]. One interpretation of our data could be that the higher expression levels of SPHK1 in the more inflamed adipose tissue of the women with fatty livers, as compared to the less inflamed adipose tissue of women with normal liver fat content, and the correlations between SPHK1 expression and ceramide/sphingomyelin concentrations in the cohort as a whole reflect a protective mechanism to counteract the potentially detrimental consequences of ceramide accumulation, but this is speculative and future studies are needed to investigate this.

Strengths of our study include the investigation of human adipose tissue biopsies from three independent patient groups and two different depots, and the availability of measures of hepatic fat content in the obese women, and hepatic gene expression data in the non-obese subjects enabling us to investigate relationships between liver and adipose tissue. A limitation is that comparisons between the different patient groups cannot be made since the groups were not anlaysed at the same time, thus only within group comparisons can be made. Another limitation is the availability of only mRNA quantification of sphingomyelinase expression rather than protein concentration or enzyme activity. Nonetheless, protein expression of sphingomyelinase was confirmed and its location in adipose tissue established by immunohistochemistry. The small size of the biopsies obtained precluded more extensive analysis. Analysis of only adipocytes was not performed since we aimed to explore the expression of ceramide-metabolising enzymes in adipose tissue as a whole (including inflammatory cells, connective tissue, blood vessels etc.) rather than exclusively in adipocytes. However, immunohistochemical analysis provided information as to the cellular location of certain proteins.

## Conclusions

Our data suggest that sphingomyelinase-mediated production of ceramide from sphingomyelin may be one mecha-nism contributing to the development of inflammation within human adipose tissue. An increased expression of SMPD3 in inflamed adipose tissue and in intra-abdominal compared to subcutaneous tissue, and its close association with adipose tissue ceramide levels might indicate an important role for this sphingomyelinase.

## Abbreviations

ACSL4: Acyl-CoA synthetase long-chain family member 4; ASAH: N-acylsphingosine amidohydrolase (ceramidase); Apo B: Apolipoprotein B; CCL2: Chemokine (C-C motif) ligand 2 (monocyte chemoattractant protein 1 MCP-1); CCL3: Chemokine (C-C motif) ligand 3; CDH5: Cadherin 5 (vascular endothelium); CERK: Ceramide kinase; CGT: Ceramide glycosyl transferase; DEGS: Dihydroceramide desaturase; DGAT2: Diacylglycerol O-acyltransferase 2; HIF-1α: Hypoxia-inducible factor 1α; LASS: LAG1 homolog (ceramide synthase); PNPLA3: Patatin-like phospholipase domain-containing protein 3 (adiponutrin); PPARG: Peroxisome proliferator activated receptor gamma; RPLP0: Ribosomal protein large P0; SELE: Selectin E; SGMS: Sphingomyelin synthase; SMPD: Sphingomyelin phosphodiesterase (sphingomyelinase); SPTLC: Serine palmitoyl transferase long-chain; TBP: TATA box binding protein; UGCG: UDP-glucose ceramide glucosyltransferase; VEGF: Vascular endothelial growth factor.

## Competing interests

The authors declare that they have no competing interests.

## Authors' contributions

MK participated in study design, data collection, statistical analysis, data interpretation and manuscript writing. JG, JW, JL, AF-C and MO participated in data collection. SAS participated in study design. HY-J participated in study design and data interpretation. PE participated in study design, data interpretation and manuscript writing. RMF conceived the study, participated in study design, statistical analysis, data interpretation and manuscript writing. All authors read and approved the final manuscript.
